# Satellite-based terrestrial production efficiency modeling

**DOI:** 10.1186/1750-0680-4-8

**Published:** 2009-09-18

**Authors:** Ian McCallum, Wolfgang Wagner, Christiane Schmullius, Anatoly Shvidenko, Michael Obersteiner, Steffen Fritz, Sten Nilsson

**Affiliations:** 1 International Institute for Applied Systems Analysis, Schlossplatz 1, A-2361, Laxenburg, Austria; 2 Institute of Photogrammetry and Remote Sensing, Technical University, Gußhausstraße 27 - 29, 1040 Vienna, Austria; 3 Institute of Geography, Friedrich-Schiller-University, Grietgasse 6, 07743 Jena, Germany

## Abstract

Production efficiency models (PEMs) are based on the theory of light use efficiency (LUE) which states that a relatively constant relationship exists between photosynthetic carbon uptake and radiation receipt at the canopy level. Challenges remain however in the application of the PEM methodology to global net primary productivity (NPP) monitoring. The objectives of this review are as follows: 1) to describe the general functioning of six PEMs (CASA; GLO-PEM; TURC; C-Fix; MOD17; and BEAMS) identified in the literature; 2) to review each model to determine potential improvements to the general PEM methodology; 3) to review the related literature on satellite-based gross primary productivity (GPP) and NPP modeling for additional possibilities for improvement; and 4) based on this review, propose items for coordinated research.

This review noted a number of possibilities for improvement to the general PEM architecture - ranging from LUE to meteorological and satellite-based inputs. Current PEMs tend to treat the globe similarly in terms of physiological and meteorological factors, often ignoring unique regional aspects. Each of the existing PEMs has developed unique methods to estimate NPP and the combination of the most successful of these could lead to improvements. It may be beneficial to develop regional PEMs that can be combined under a global framework. The results of this review suggest the creation of a hybrid PEM could bring about a significant enhancement to the PEM methodology and thus terrestrial carbon flux modeling.

Key items topping the PEM research agenda identified in this review include the following: LUE should not be assumed constant, but should vary by plant functional type (PFT) or photosynthetic pathway; evidence is mounting that PEMs should consider incorporating diffuse radiation; continue to pursue relationships between satellite-derived variables and LUE, GPP and autotrophic respiration (Ra); there is an urgent need for satellite-based biomass measurements to improve Ra estimation; and satellite-based soil moisture data could improve determination of soil water stress.

## Introduction

Carbon is removed from the atmosphere via photosynthesis by plants. Upon entering the terrestrial ecosystem it is termed gross primary productivity (GPP), with the difference between carbon gain via GPP and carbon loss through plant respiration defined as net primary productivity (NPP) [1]. At the regional or global scale, carbon fluxes (i.e. NPP) cannot be directly observed [2]. NPP is difficult to measure (in-situ) over large areas owing to spatial variability of environmental conditions and limitations in the accuracy of allometric equations [3]. Therefore, a variety of methods have been developed to estimate carbon fluxes, including flux towers e.g. [4], carbon accounting e.g. [5], global vegetation models e.g. [6], atmospheric measurements e.g. [7] and satellite-based techniques e.g. [8].

Among all these methods, only satellite observations provide globally consistent, spatially highly resolved observations of numerous surface variables that affect carbon exchanges [9]. However, models are required which can ingest this raw information and convert it into fluxes. Their interpretation of the underlying biochemical, biophysical and 3-D geometric properties of vegetation and soils is the main challenge in the application of satellite-based earth observation data for modeling the terrestrial carbon cycle [10].

Production efficiency models (PEM), sometimes referred to as diagnostic models, have been developed to monitor primary production, taking advantage of available satellite data. PEMs combine the meteorological constraint of available sunlight reaching a site with the ecological constraint of the amount of leaf-area absorbing that solar energy, avoiding many complexities of carbon balance theory [11]. PEMs are based on the theory of light use efficiency (LUE) which states that a relatively constant relationship exists between photosynthetic carbon uptake and radiation receipt at the canopy level [12]. In addition to LUE, PEMs typically require inputs of meteorological data (i.e. radiation, temperature and others) and the satellite-derived fraction of absorbed photosynthetically available radiation (FAPAR).

PEMs complement the many ecophysiological process models that simulate carbon exchange [13]. A model comparison of 17 global NPP models featured several PEMs whose results compared well with process models [2]. Currently two PEMs are producing NPP operationally at the global scale, namely C-fix [14] and MOD17 [8]. Challenges remain however in the application of the PEM methodology to global NPP monitoring. In particular, determination of LUE [15,16] and autotrophic respiration [17] remain somewhat uncertain. Additional uncertainties have been identified in the meteorological data [18] and in the biophysical data [19], both key components in PEMs. Several recent studies suggest that simple regressions between GPP and remote sensing products might yield better results than those incorporating meteorological data [20]. All of these issues point to the need for a review of the current state of PEMs.

A variety of excellent reviews have addressed various aspects of PEMs in recent years: [2,3,21-24], however none have specifically reviewed the existing published models. The objectives of this review are as follows: 1) to describe the general functioning of six PEMs (CASA; GLO-PEM; TURC; C-Fix; MOD17; and BEAMS) identified in the literature; 2) to review each model to determine potential improvements to the general PEM methodology; 3) to review the related literature on satellite-based GPP and NPP modeling for additional possibilities for improvement; and 4) based on this review, propose items for coordinated research.

## Production Efficiency Modeling

### Background

Photosynthesis by plants provides the carbon and energy that drives most biological processes in ecosystems. Similar to photosynthesis by individual leaves, GPP varies diurnally and seasonally in response to changes in light, temperature, water and nitrogen supply while differences among ecosystems in annual GPP are determined primarily by the quantity of leaf area and the length of time that this leaf area is photosynthetically active [1]. While the relationship between photosynthesis and irradiance can be markedly non-linear for individual leaves, it approaches linearity at the canopy level, presumably because a smaller fraction of leaf area is operating under light-saturated conditions [12,25].

In 1953, the first steps were taken to calculate productivity of an entire plant community indirectly on the basis of light [26]. However, Monteith [27,28] is commonly credited with first proposing the existence of a conservative (linear) relationship between the rate of NPP and the rate at which solar energy is absorbed by the foliage, conducting experiments with crop species during the vegetative stages of growth under optimal growing conditions. The ratio between these two quantities has been called the conversion efficiency of absorbed radiation into dry matter, and was used in many simple models of crop growth, i.e. bypassing the complex process of photosynthesis and respiration known to depend on many environmental variables [21]. In crop canopies, where water and nutrients are highly available, the linear relationship between canopy carbon exchange and irradiance extends up to irradiance typical of full sunlight [1]. However in forest canopies, the relationship is not so simple and LUE is dependent upon other factors. An increasing number of studies indicate that LUE fluctuates among vegetation species, stand age, soil fertility, etc [15,16,29]. It was noted, however, that light absorption and utilization are decoupled so that convergence is to be expected on gross production rather than net production, owing to differences in respiratory costs associated with synthesis and maintenance of plant constituents and associated 'payback intervals' on carbon investment in different functional types [3].

An attractive feature of the PEM concept is its suitability for use with remotely sensed observations [30], which provide both the timing of the active period and the quantitative values of FAPAR. The approximation that the annual photosynthetic activity is a conservative function of APAR permits monitoring of biospheric activity with little need for ancillary information [2]. While functional convergence provides a basis for the use of remote sensing of light absorption in measurement of primary production, models driven with light absorption must also include terms that describe the actual respiratory costs of maintenance and synthesis [3].

"Modern PEMs", however, should not be confused with early experimental models based solely on correlation relationships between spectral vegetation indices and crop yield [31]. They are now generally global, depend heavily on satellite and meteorological datasets and operate at high spatial and temporal resolution. They typically consider GPP and NPP separately and contain terms to describe plant respiration. A chronology of modeling efforts claims the first global PEM (CASA) appeared in 1993 [32].

### PEM Algorithm

In general, all PEMs employ a similar basic methodology to calculate NPP. Typically this involves two steps, first calculating GPP (Equation 1) and then subtracting Autotrophic Respiration (Ra) (Equation 2) to derive NPP. Variation among the different methods generally appears in the determination of LUE, the use of scalars and Ra. Timesteps range from daily to yearly and spatial resolution from 1 km to 1 degree.

(1)

(2)

where:

GPP   Gross Primary Productivity (g C m^2^)

PAR   Photosynthetically Active Radiation (MJ m^2^)

FAPAR   Fraction of Absorbed PAR (dimensionless %)

LUE   Light Use Efficiency (g C MJ^-1^)

Scalars   Temperature, (VPD) Vapour Pressure Deficit, etc (0-1)

NPP   Net Primary Production (g C m^2^)

Ra   Autotrophic respiration (g C m^2^)

#### PAR

Photosynthetically active radiation (PAR) is the solar radiation reaching the canopy in the wavelength region of visible light (0.4 - 0.7 micrometers). This is typically derived from meteorological datasets, but may also come from satellite products [33]. At the global level, PAR is comprised of roughly equal amounts of direct (clear sky) and diffuse (cloudy, aerosols) radiation, while at the regional level large differences occur. Of crucial importance is the geometry of the incoming sunlight, which is comprised of direct and diffuse components [34-37].

#### FAPAR

The fraction of absorbed PAR (FAPAR) is defined as the fraction of PAR absorbed by green vegetation. FAPAR is difficult to measure directly, but is inferred from models describing the transfer of solar radiation in plant canopies, using remote sensing observations as constraints [25,38,39]. Comparisons between the actual FAPAR products derived by the various space agencies or projects reveal discrepancies: they are mainly due to the different strategies in the retrieval methodologies but also to the quality of input variables [38].

#### LUE

Light use efficiency (LUE) is typically defined in biology as the ratio between accumulated biomass and PAR (sometimes referred to as radiation use efficiency (RUE), a similar ratio but based on total solar radiation intercepted). LUE can be defined as measured on the basis of gross production, net production, environmentally stressed or hypothetically unstressed (i.e. maximum) production [40]. Difficulties arise with the lack of a universally agreed upon definition of LUE, a quotient where the numerator quantifies production and the denominator irradiance [41]. Historically, the numerator is either NPP (aboveground or total) or GPP, while incident, intercepted or absorbed total shortwave or PAR have been used as denominators. Literature derived LUE generally corresponds to above-ground LUE [42].

The conversion of absorbed radiation into dry matter can be computed from a variety of approaches: a constant 'conversion efficiency' or the product of an optimum value by other factors representing environmental stresses [42]. In most PEMs the potential (maximum) LUE value is empirically derived, then reduced due to environmental constraints [2].

#### Scalars

Scalars representing environmental constraints are typically meteorologically derived (but may also be satellite-based) variables that serve to reduce the LUE value at a specific time and location due to predicted plant stress, e.g. high vapour pressure deficits (VPDs) have been shown to induce stomatal closure in many species, while low temperatures inhibit photosynthesis. Depending upon the PEM, scalars such as temperature, VPD and soil moisture are used to reduce the maximum LUE values, e.g. through linear ramp functions [43].

#### Ra

Autotrophic plant respiration (Ra) is a large, environmentally sensitive component of the ecosystem carbon balance, and net ecosystem carbon flux will change as the balance between photosynthesis and respiration changes [3,44,45]. Autotrophic respiration describes the respiration released from living plant tissues, including leaves, roots and wood. Plant respiration can be separated into three separate components: growth respiration; maintenance respiration and the respiratory cost of ion uptake - with modeling studies indicating that Ra is about half (48-60%) of GPP when a wide range of ecosystems are compared [1]. Ra is handled differently in each of the models, ranging from a simple linear function of temperature to empirical methods.

### Model Descriptions

A review was made of the key attributes and results of six published PEMs (Table 1). A brief description of the unique properties of the six PEMs is given below.

**Table 1:** Attributes and results of six global PEMs available from the literature.

***PEM***	***Study period***	***Timestep***	***Cell-size***	***LUE Scalars***	***LUE-GPP*** **(*g C MJ*^-1^**)	***NPP*** **(*Pg C yr*^-1^**)	***Reference***
**CASA**	1982-1998	Month	0.5°	T, AET, PET	0.39^e^	48.0^c^	[46]
**GLO-PEM**	1981-2000	10 days	8 km	T, SW, VPD	1.03-1.64^a^	69.7^b^	[48]
**TURC**	1998	Month	1°	No Scalars	1.10	64.0	[49]
**C-Fix**	1998-2008	10 days	1 km	T, CO_2_, SW, EF	1.10	NA^f^	[14]
**MOD17**	2000-2008	Day/Year	1 km	T, VPD	0.68-1.159	56.0^d^	[18]
**BEAMS**	1982-2000	Month	1°	T, h, SW	0.0-1.0		[53]

^a ^[13]; ^b ^[31]; ^c ^[78]; ^d ^[51]; ^e ^based on NPP; ^f^NA (globally not available in published literature)

T Temperature; SW Soil Water; VPD Vapour Pressure Deficit; AET Actual Evapotranspiration; PET Potential Evapotranspiration; CO_2 _fertilization factor; EF Evaporative Fraction; h Relative Humidity

#### CASA

The Carnegie Ames Standford Approach (CASA) is a numerical model of monthly fluxes of water, carbon and nitrogen in terrestrial ecosystems. Estimates of terrestrial NPP fluxes depend on inputs of global satellite observations for land surface properties and on gridded model drivers from interpolated weather station records [46]. LUE is set uniformly at 0.39 g C MJ^-1 ^PAR, a value that derives from calibration of predicted annual NPP to previous field estimates. This model calibration has been assessed globally [47]. Temperature stress is computed with reference to derivation of optimal temperatures for plant production. CASA includes a water stress scalar estimated from monthly water deficits, based on a comparison of moisture supply to potential evapotranspiration demand [47]. This is the only model that does not separately calculate GPP. Instead it models NPP directly, thus avoiding a Ra calculation.

#### GLO-PEM

The Global Production Efficiency Model (GLO-PEM) consists of linked components that describe the processes of canopy radiation absorption, utilization, autotrophic respiration, and the regulation of these processes by environmental factors [48]. It was designed to run with both biological and environmental variables derived entirely from satellites and is thus unique as it is the only PEM to do so (except for distinguishing between C3 and C4 vegetation). The portion of C3 or C4 vegetation per pixel is calculated as a function of above ground biomass (calculated from the minimum annual visible channel reflectance [3]) and air temperature. In contrast to other modern PEMs, GLO-PEM estimates LUE rather than prescribing values based on limited field observations [31]. LUE is reduced by environmental factors that control stomatal conductance i.e. the effects of air temperature, VPD and soil moisture [48].

Autotrophic respiration is modeled for maintenance respiration using a semi-empirical relationship as a function of vegetation, biomass, air temperature and photosynthetic rate, while growth respiration is a constant of GPP (0.25). Below-ground biomass is not estimated, thus Ra is assumed to apply to the whole plant [31].

#### TURC

When first published, the main originality of the model Terrestrial Uptake and Release of Carbon (TURC) was to relate light absorption to GPP (rather than to NPP), and to derive parameters from CO_2 _exchange measurement (canopy fluxes for photosynthesis, chamber measurements for respiration) [49]. Originally, LUE was derived empirically (1.10 g C MJ^-1^) and used to calculate GPP with environmental constraints applied to Ra [49]. Frost stress on photosynthesis was later included by reducing the conversion efficiency by 50% during the three days following a severe frost, defined by a daily mean air temperature lower than -2°C. Unique LUE values were also used for high latitude wetlands, which proved to be substantially reduced from non-wetlands and reduced the maximum LUE value based on values of low mean annual temperature [49].

Autotrophic respiration in TURC is the sum of maintenance (leaves, fine roots and wood) and growth respiration (a constant fraction (0.28) of GPP minus maintenance respiration). An average maintenance respiration coefficient at 20°C has been determined for each organ (using experimental data). Maintenance respiration is then scaled as a linear function of temperature and organ biomass. A vegetation map and normalized difference vegetation index (NDVI) data are used to estimate biomass for each cell [49].

#### C-Fix

The parametric PEM C-Fix, estimates carbon mass fluxes from local to global scales [14]. C-Fix is operational, providing global NPP since 1998. For a given point location, the original model estimates carbon fluxes on a daily basis. C-Fix is a mass balance model based on the parameterization of FAPAR derived from remotely sensed NDVI [14]. RUE is set equal to 1.10 g C MJ^-1^. This is reduced by the normalized temperature dependency factor and the normalized CO_2 _fertilization factor. Further refinements were introduced to C-Fix, namely integration of a water limitation; temperature buffering and estimates of soil temperature [14].

In C-Fix, the autotrophic respiration reduction factor is modeled as a simple linear function of daily mean atmospheric air temperature. This parametric model for respiratory losses is assumed state (phytomass) independent [50]. The dependency of maintenance respiration on the amount of living biomass is neglected.

#### MOD17

The Moderate Resolution Spectroradiometer (MODIS) sensor has provided near real-time global estimates of GPP and annual NPP (MOD17) since March 2000, on an operational basis. One of the largest assumptions made (to implement MOD17 globally) is the use of a constant maximum RUE within each of the 12 biomes used [18]. A minimum temperature scalar reduces the conversion efficiency when cold temperatures limit plant function. The MOD17 GPP algorithm does not have a winter dormancy function to regulate winter productivity [18]. As a global generalisation, the algorithm truncates GPP on days when the minimum temperature is below 0°C [43]. A scalar is used to reduce the maximum conversion efficiency when the VPD is high enough to inhibit photosynthesis. The effect of soil water availability is not included in the GPP algorithm [18]. To partially account for this issue, sensitivity to VPD is increased in the model as a surrogate for drought effects. The model is parameterized with eddy covariance data.

In MOD17, maintenance respiration by leaves and fine roots is subtracted from GPP (on a daily basis). Annual NPP is then calculated by subtracting maintenance respiration by all other living parts except leaves and fine roots (e.g. livewood) and growth respiration [51]. Maintenance respiration and growth respiration components are derived from allometric relationships linking daily biomass (leaf biomass is calculated using leaf area index (LAI) and specific leaf area defined for each plant functional type (PFT) [43]) and annual growth of plant tissues to satellite-derived estimates of leaf area index.

#### BEAMS

The Biosphere model integrating Eco-physiological And Mechanistic approaches using Satellite data (BEAMS) is a diagnostic model requiring both satellite and climate data [52]. It includes a carbon cycle submodel to capture GPP and autotrophic respiration [53]. GPP was modeled based on the LUE concept using satellite-based monthly FAPAR data and a stress calculation which considered air temperature, relative humidity, soil moisture and atmospheric CO_2 _concentrations. GPP is allocated into leaf, stem and root components by an empirical equation using climate parameters.

In BEAMS, the Ra of leaves, stems and roots consists of maintenance and growth respiration. Maintenance respiration is modeled in proportion to biomass (see MOD17) with temperature dependence (Q_10 _= 2), while growth respiration is modeled in proportion to the potential NPP [53].

## Error Sources and Variability in PEMs

A variety of attempts at evaluation of PEMs have been published, most commonly at the global scale in the form of inter-comparison studies e.g. [2,42,53,54] or over the data-rich areas of North America e.g. [18] and Europe e.g. [14,55] with in-situ measurements. However, determining the uncertainty of carbon fluxes is difficult and requires understanding of the uncertainties of model structure, data and model parameter uncertainties and particularly the temporal and spatial inaccuracy of the input data retrieval [56].

Early attempts at NPP modeling resulted in values for global NPP of approximately 60 Pg C yr^-1 ^[57]. An inter-comparison of global NPP models found values in the range of 40 - 80 Pg C yr^-1^, with results from PEMs fitting well within this value [2]. However, while it appears that between model variability is relatively low, within model variability is likely much higher (more so at a regional scale). Models that agree on the value of certain outputs (e.g. annual NPP) may disagree on the underlying processes (e.g. differences in rates of photosynthesis vs. plant respiration) [42]. See Table 2 for a selection of PEM error sources and variability.

**Table 2:** A selection of published error sources and variability from various input variables used in PEMs.

**Dataset**	**Error Sources/Variability**	**Citation**
**Meteorology**	16 - 43% difference in NPP	[58]
	28% difference in GPP	[18]
**PAR**	35-62% over predicted NPP	[107]
	13% difference in NPP	[33]
**LUE**	0.2 - 1.8 g C MJ^-1 ^(in-situ)	[3]
**FAPAR**	RMSE 0.1 - 0.12	[108]
	8-20% greater than in-situ	[109]

Too few studies exist that measure uncertainties in carbon flux modeling and remotely sensed data assimilation. However, error propagation and Monte-Carlo approaches to assess uncertainty in a PEM are available [56]. Additionally, a sensitivity analysis to assess which climate variables most influenced simulation differences of a PEM, via three climate datasets, found large differences [58]. Certainly, a more severe test of the models, including comparison with observed ecosystem fluxes at tower sites and with models driven by site meteorology, needs to be pursed [55].

## Key Research Items

The following sections describe various shortcomings and potential improvements to the PEM methodology based on a review of the six models in this study (CASA; GLO-PEM; TURC; C-Fix; MOD17; and BEAMS) and related studies, along with suggested key research items.

### Light Use Efficiency

Estimates of LUE are generally not well constrained and provide a large source of error in model estimates of global NPP, arising from different philosophies on the environmental and biological controls of LUE and the methods adopted to estimate this parameter [15]. One major difference between models is the use of a constant versus biome, PFT or photosynthetic pathway specific LUE. Various studies suggest that LUE varies with factors such as forest stand age, species composition, soil fertility, foliar nutrients, drought, radiation, phenological stage, climatic condition, temperature, and others [3,16,29,49,59]. Therefore any model that incorporates the premise that LUE is constant can only be considered approximate and will be increasingly in error over shorter periods [40].

An understanding of the factors that control the efficiency with which forest canopies harvest available light to fix carbon via photosynthesis is necessary for the development of useful PEMs [29]. High LUE was found in boreal regions and in the northern hemisphere tropics [15]. Within boreal zones, Eurasian LUE is higher than North American LUE and has a distinctly different seasonal profile. More work is needed on the LUE of forested wetlands with different species mixtures [16], with low LUE found in high latitude wetlands [49]. In addition, LUE differed significantly among forest cover types and between years [16]. To date, there has been little work to account for variations in LUE introduced by herbivory, disease and differences in respiratory costs [3] although a benefit of using satellite data means that to some extent these elements are accounted for.

For large areas, in which the vegetation cover, LAI, physiognomy, and species are likely to be heterogeneous, field-plot scale empirical derivation of LUE is not appropriate [60]. However, it was suggested for model improvement, to selectively alter values for maximum LUE based on observations at eddy covariance flux towers [17]. This may work for areas with high sampling frequency, but for much of the globe will be too infrequent. Current efforts attempt to derive LUE directly from satellites [61-65] (see Recent Advances). Regular direct measurements of LUE would make it possible to capture the real variation of photosynthetic efficiency and then to assimilate it into PEMs [64].

#### Light Use Efficiency: key findings/research items

• LUE should not be assumed constant, but should vary by PFTs e.g. [18], photosynthetic pathway e.g. [48], or other means. LUE is most relevant in the context of GPP rather than NPP, due to differences in respiration among PFTs [3,13,31]

• More empirical studies are required to determine LUE under various environmental conditions - i.e. create a global publicly available database e.g. [22]

• Intensify efforts to derive LUE directly from satellites e.g. [61-65]

### Biophysical, Meteorological and Atmospheric Variables

Only one of the six published PEMs in this review (GLO-PEM) relied purely on satellite-derived meteorological measurements - the rest have utilized observation-based meteorological datasets. The single largest error associated with GPP at most sites likely derives from meteorology, with a 28% difference between GPP generated from climatology data versus tower data [18]. The largest differences in temperature sensitivity among NPP estimates occurred in the northern latitudes [54].

Water stress is one of the primary limiting functions controlling photosynthesis by terrestrial ecosystems [66]. Under-estimation of VPD contributes greatly to overestimation of GPP [18]. High VPDs > 2000 Pa, have been shown to induce stomatal closure in many species. This level of daily atmospheric water deficit is commonly reached in semi-arid regions of the world for much of the growing season [43].

Additionally, models that do not directly account for soil water stress may be problematic at water-limited sites [18]. The accuracy of NPP estimates for forests more affected by soil water conditions than geographical moisture conditions, can be improved by using a soil water index [59]. The strong impact of soil moisture on the European carbon balance was demonstrated using a PEM [67]. Even if the water status of the leaf remains unchanged, stomatal conductance decreases with decreasing soil moisture [48]. Additionally, forest growth in high latitudes is not only limited by temperature, radiation and nutrient availability but also by the availability of liquid soil water, and it was, therefore, recommended to include permafrost in models [68]. In permafrost regions, a large amount of water is lost as runoff during spring and is hence not available for vegetation later in the growing season [68].

Accurate estimates of NPP are also highly dependent upon the quality of the global daily estimates of PAR [11]. A comparison of PAR products found biases and rms errors > 25% [33]. The importance of FAPAR data in model-data driven productivity estimation methods based on the PEM approach was also noted [19], especially as FAPAR is often the only satellite-based variable used in PEMs.

Three of the models reviewed here (GLO-PEM, C-Fix and BEAMS) account for CO_2_. Elevated CO_2 _increases both water use efficiency and RUE, even when those resources are at low-growth restrictive levels [69]. CO_2 _fertilization is important for plant growth activity, and needs to be accounted for in satellite-based NPP models [53].

#### Biophysical, Meteorological and Atmospheric Variables: key findings/research items

• For both biophysical and meteorological variables a variety of datasets are available - here it would be beneficial to set standards in terms of input datasets, thus allowing for easier inter-model comparison

• Further investigation into meteorological remote sensing products such as land surface air temperature which can potentially be used as a measure of both temperature and VPD e.g. [70]

• Soil moisture available from satellite measurements e.g. [71], should be considered for inclusion in PEMs e.g. [67]. Additionally, regional effects of permafrost should be considered e.g. [68]

• CO_2 _fertilization is important for plant growth activity, and needs to be accounted for in satellite-based NPP models [53]

### Diffuse Radiation

Substantial evidence exists that the solar irradiance incident at the surface has declined substantially over the last 50 years (with a potential increase in diffuse radiation), pointing to the inclusion of diffuse radiation in models [72]. At the global level, PAR is comprised of roughly equal amounts of direct (clear sky) and diffuse (cloudy, aerosols) radiation, while at the regional level large differences occur. Diffuse and direct radiation differ in the way they transfer through plant canopies and affect the summation of non-linear processes like photosynthesis differently than what would occur at the leaf scale [35]. For example, conifer needles are particularly effective in absorbing diffuse light, which provides a more uniform illumination of the overall canopy.

Diffuse radiation results in higher LUE, with GPP dependent upon the composition of incident irradiation (the ratio of diffuse to direct light) [35,61]. LUE was found to be highest on overcast days and decreased on clear-sky days [36,37]. The relationship appears to be quite general, although the magnitude of the effect is related to the structural properties of the canopy and the productive capacity of the vegetation [37]. To the contrary, others consistently recorded a decrease in primary productivity, owing to the decline in total irradiance that occurs when clouds obscure the solar dish [73]. Additionally, current LUE approaches fail to predict GPP in a tropical rain forest as they neglect GPP saturation when radiation is high [74]. In general, systems adapted to large amounts of diffuse light (e.g. boreal) don't do well under high levels of direct light (and subsequent high VPD) and vice versa. To some extent, temporal integration gets around these issues that are most pronounced at short time scales.

It is a challenge for the next generation of production models - which rely on LUE, to develop new algorithms to accommodate diffuse radiation [35]. It was suggested that the inclusion of estimates of diffuse radiation as a scalar for LUE will substantially improve estimates of gross photosynthesis from PEMs, especially at daily time resolution [29]. An alternative formulation of the GPP algorithm was envisioned that specified a different maximum LUE under clear sky and overcast conditions, then ranged between those values depending on the degree of cloudiness [36].

#### Diffuse Radiation: key findings/research items

• Evidence is mounting that PEMs should consider incorporating diffuse radiation, especially at daily resolution e.g. [29,35-37], however caution should be applied e.g. [73]

• Several published methods which already incorporate diffuse light in PEMs should be further investigated e.g. [72,75]

• PEMs should also consider the need to account for GPP saturation when radiation is high e.g. [74]

### Phenology

Phenology is to a certain extent captured in the current PEM models via the meteorological data and FAPAR. However, the timing of snowmelt and soil thaw, the onset of warming in the spring and other factors affecting phenology can have a large impact on the annual carbon balance in forests, in particular in cold climates [76,77]. Early in the season, efficiency may be reduced by the expense of leaf and fine root construction, while late in the season it may be reduced while transferring leaf metabolites into other tissues. For boreal systems there is an observed lag between the time temperatures permit photosynthesis and the time the photosynthetic machinery becomes active [78]. Seasonally, summer estimates of GPP are closest to tower data while spring estimates are the worst, most likely the result of the relatively rapid onset of leaf-out [18].

During winter and early spring, evergreen boreal conifers are severely stressed as light energy cannot be used when photosynthesis is preempted by low ambient temperatures. Severe intermittent low-temperature episodes during this period actually reversed physiological recovery [79]. Night frosts depress photosynthesis the following day and the effect of severe frost is visible for several days [80]. Frost stress on photosynthesis is included in the TURC model by reducing the conversion efficiency by 50% during the three days following a severe frost, defined by a daily mean air temperature lower than -2°C [49]. As a global generalisation, the MOD17 algorithm truncates GPP on days when the minimum temperature is below 0°C [43].

The introduction of new techniques to better capture the start and finish of the growing season may improve a PEMs ability to detect these crucial transition periods. A method was developed using space-borne scatterometer measurements to detect the onset of spring thaw and the freeze/thaw cycle duration, based on the significance of diurnal differences with respect to long-term noise [81]. In general, backscatter is high and relatively stable during winter. During spring melt, however, rapid fluctuation is observed and only after the thaw does backscatter stabilize, albeit at a lower level. The onset of the spring thaw period coincides with the first days of increased CO_2 _fluxes above the late winter baseline. The end of daily freeze thaw cycles corresponds to the switch from source to sink in evergreen boreal forest [81].

#### Phenology: key findings/research items

• Investigate incorporating scatterometer data to account for spring thaw and the freeze/thaw cycle duration e.g. [81]. This data could be used to prevent assigning carbon uptake too early in the spring due to e.g. rising FAPAR values

• Consider some form of frost stress, perhaps via air temperature e.g. [49], if not already included

### Vegetation Morphology

Among the PEMs, land cover is currently only used in MOD17 for the assignment of LUE. However, this was noted as a potential source of error because of landscape heterogeneity at the sub pixel scale [18], leading to overestimation of GPP in complex ecosystems. Additionally, large discrepancies occur among land cover datasets, and choice of one dataset over another will affect model outcome [82,83]. Vegetation related disturbances should however be adequately represented e.g. [84], with various satellite-based options available [85]. It may be, however, that FAPAR is capturing this adequately - perhaps crucial only at shorter timescales.

An important detail which could introduce uncertainty into PEMs is the fact that they generally consider whole forest stands via the notion of convergence, largely ignoring canopy layers. In some high latitude regions for certain species, forest understory and a green forest floor can generate up to 50% of total NPP [86].

Several authors have suggested that leaf area index (LAI) is the principal scaling variable for both gross photosynthesis and ecosystem respiration of northern deciduous and coniferous forests e.g. [76]. Problems exist however with the quality of global satellite-derived LAI products [87], currently making this a difficult variable to include in PEMs. Saturation at high LAI values together with biases due to soil reflectance, vegetation clumping and others have limited performance [88].

#### Vegetation Morphology: key findings/research items

• Exercise caution if utilizing land cover products or LAI in PEMs [18,82,87]

• Consider methods to account for disturbance effects on vegetation morphology e.g. [85]

### Autotrophic Respiration

Plant respiratory regulation is too complex for a mechanistic representation in current terrestrial productivity models [89]. Of the six PEMs compared in this review, all but one separately account for autotrophic respiration (Ra), needed to convert GPP into NPP. All but one of these define Ra as the sum of growth and maintenance respiration, estimating Ra for leaves, wood and roots. Variation in maintenance respiration is the most likely cause for variability in the efficiency of converting GPP into NPP. According to [17], over prediction in NPP is a problem of underestimating Ra rather than overestimating GPP.

PEMs that assume maintenance respiration is dependent upon the amount of living biomass obviously require some measure of biomass. Previous efforts have relied on correlations of biomass with optical reflectance measurements. However, forest biomass is poorly quantified across most parts of the planet [90]. Owing to the difficulty in estimating above ground wood (of live trees), it was suggested for forests, to make stemwood Ra a fixed proportion of total Ra.

Work is ongoing to determine whether autotrophic respiration can be estimated from remote sensing data alone [91]. For densely forested sites, respiration is strongly related to land surface temperature (LST), with relatively little variation in this relationship between sites [92].

#### Autotrophic Respiration: key findings/research items

• There is an urgent need for satellite-based biomass measurements to improve Ra estimation - efforts such as BIOMASS [93] and various RADAR and LiDAR [94] research efforts could be applied here

• Pursue studies attempting to link plant respiration to satellite-derived variables e.g. [91]

• The research community could benefit from a comprehensive literature review specifically focused on autotrophic respiration modeling with satellite data.

### Recent Advances

New methods are currently under development which will perhaps enhance or replace the PEM methodology in the future; however at present these are largely not operational. The general trend is to develop new methods from satellite-based tools that measure LUE or GPP directly. Several proposed (presently unsupported) satellite missions from the European Space Agency (ESA) (i.e. FLEX and ASCOPE) [93] specifically target this issue. In addition, the GOSAT  and OCO  missions will provide global measurements of CO_2 _fluxes which could be used to help calibrate and/or validate PEMs.

The use of chlorophyll content measured from satellite to predict crop productivity was proposed; however variation in GPP due to short term stress cannot be detected [61]. It was therefore recommended to combine other products along with the use of a red-edge. A continuous-field LUE retrieved from satellite data using the photochemical reflectance index (PRI) was developed [95]. Indices such as the PRI and the enhanced vegetation index (EVI) have been shown to correlate with LUE, however the relationship between LUE and PRI varies considerably between vegetation types and years [63,64]; and LUE and EVI are not well correlated for evergreen sites. The relationship between PRI and LUE improves when the analysis is restricted to small changes of viewing angles [65]. Combining the LST product from MODIS with the EVI for 16-day means, [91] found an improved correlation to flux tower GPP data for 11 sites as compared to MOD17.

Replacing the LUE approach with a more general PAR-response approach was advocated by [74] - one that includes common response features of vegetation canopies to environmental conditions (particularly light saturation), based on work in a tropical biome. Another approach was presented to estimate GPP using FAPAR in conjunction with GPP estimates from eddy covariance measurements, suggesting that use of simple regression between GPP and a remote sensing product yield more robust results than models additionally based on meteorological input [20]. In a related study, flux data were used to constrain and parameterize a neural network structure using a limited number of driving variables to estimate spatial and temporal carbon fluxes for European forests [96]. Additionally, hyperspectral remote sensing offers the possibility of sensing changes in the xanthophyll cycle and fluorescence, both related to photosynthesis [64].

#### Recent Advances: key findings/research items

• Continue to pursue relationships between satellite-derived variables and LUE or GPP e.g. [64,91]

• Results from proposed satellite missions i.e. FLEX and ASCOPE [93] could improve the capability to model productivity from space - these efforts should be pursued

• Results from the GOSAT/OCO missions should be used to improve validation efforts by comparison with PEM outputs - a potential improvement over the current sparse network of FLUXNET [97] towers currently used for validation

## Discussion

Progress in PEMs and related development has been steady since the earliest models were proposed [26,30]. The first global PEMs utilizing satellite data appeared in the early 1990s [32] i.e. CASA, GLO-PEM and TURC. Some of these models were already quite sophisticated (i.e. GLO-PEM derived the majority of its inputs from satellite data), and they have continued to be improved and updated e.g. [48]. Recently, a new generation of PEMs has emerged (i.e., C-Fix, MOD17 and BEAMS) of which C-Fix and MOD17 are operational. They incorporate generally higher resolution (spatial and temporal) data, or are more comprehensive in terms of input requirements (i.e. BEAMS). Operational models must however remain at a moderate level of complexity in order to be practical. Additionally, many regional models exist which can be used to test new ideas e.g. [72] for potential inclusion into global PEMs. Furthermore, a lot of effort among the research community is resulting in different satellite-based techniques which could be utilized in the PEM approach i.e. LST, EVI, PRI, etc.

This review aims to examine existing global PEMs and related literature in an attempt to extract key elements from each of the models to establish a proposed framework for coordinated research. Several reviews have addressed various aspects of PEMs in recent years, e.g. [3,21-24] although none have specifically reviewed the existing published models. The earliest reviews of global PEMs involved inter-comparison studies of NPP models (i.e. CASA, TURC and GLO-PEM) [2,42,54]. More recently, a comparison was made of BEAMS, MOD17 and CASA [53]. Additionally, the carbon sink archives , were designed for inter-comparison of terrestrial carbon models and include three PEMs (GLO-PEM, BEAMS and MOD17).

Although the focus of this study was on primary productivity, the ultimate goal of carbon flux modeling is to estimate net ecosystem productivity (NEP), the central term used to describe imbalances in carbon uptake and loss by ecosystems [98]. NEP is typically defined as NPP less heterotrophic respiration (Rh). Rh is generally known to be difficult to model, because it depends upon many interacting factors in the soil, such as soil carbon content, soil humidity, soil pH, soil oxidation potential, soil temperature and the micro-fauna and flora activity of the soil [50].

Of the six PEMs discussed in this review, four produce estimates of global NEP (i.e. CASA, TURC, C-Fix and BEAMS):

• CASA has a similar structure to the CENTURY [99] model, accounting for the soil profile, production and decomposition

• In TURC, Rh is related to soil temperature through a Q_10 _relationship (Q_10 _= 2) [49]. Soil moisture impact on the decomposition rate follows the CENTURY model

• C-Fix accounts for the impact of temperature on soil respiration, using a temperature dependency factor and a site-specific rate constant (based on flux measurements) [100]

• BEAMS parameterizes soil decomposition as a function of soil temperature and water content i.e. CENTURY

Soil respiration is often modeled as a simple Q_10 _or Arrhenius type function of temperature, sometimes modified by a water scalar e.g. [101]. More recently, soil respiration was modeled using a temperature, precipitation and LAI model, providing compatibility with remote sensing approaches [102]. However, prior to global application the approach needs to be tested in boreal, cold-temperate and tropical biomes, as well as for non-woody vegetation. Additionally, research suggests that differences between the apparent and intrinsic temperature sensitivity of soil respiration may be due to a correlation between soil respiration and photosynthetic rates (i.e. GPP) [103], offering another possibility for remote sensing based solutions. Research into these methods should continue with the aim of further linking soil respiration and remote sensing measurements.

## Conclusion

Since the influential work of Monteith [30], founded on the relationship between the rate of NPP and the rate at which solar energy is absorbed by foliage, the application of satellite-based PEMs for NPP monitoring has consistently evolved. With constant advances in satellite-based measurements, in-situ methods and computational ability, the PEM methodology has been refined and now delivers operational measurements of global terrestrial primary productivity at high temporal and spatial resolution. Simplification of the estimation of LUE enables remote sensing to utilize a robust approach, based in evolutionary ecology, while exploring the key advantage of a spatially and temporally contiguous monitoring capability [3].

A review of six global PEMs available in the literature (CASA; GLO-PEM; TURC; C-Fix; MOD17; and BEAMS) revealed the use of a similar conceptual framework based on the LUE methodology. However, review of the approaches and screening of the related literature have identified potential improvements that could be implemented to enhance the results of existing or new PEMs. Based upon this review, key research items were identified that appear crucial to improve the PEM methodology, including:

• LUE should not be assumed constant, but should vary by PFTs e.g. [18], photosynthetic pathway e.g. [48], or other means

• Continue to pursue relationships between satellite-derived variables and LUE or GPP e.g. [64,91]

• Evidence is mounting that PEMs should consider incorporating diffuse radiation, especially at daily resolution e.g. [29,35-37], however caution should be applied e.g. [73]

• Exercise caution if utilizing land cover products or LAI in PEMs [18,82,87]

• There is an urgent need for satellite-based biomass measurements to improve autotrophic respiration estimation

• Investigate incorporating scatterometer data to account for spring thaw and the freeze/thaw cycle duration e.g. [81]

• Soil moisture available from satellite measurements e.g. [71], should be considered for inclusion in PEMs e.g. [67]

The results of this review and the above indicated key research items suggest the creation of a global hybrid PEM could bring about a significant improvement to the PEM methodology and thus terrestrial carbon flux modeling. Each of the six PEMs reviewed apply somewhat different techniques to determine NPP. Based on this review, it is possible to identify certain features of some of the models which, if combined into a hybrid PEM, could potentially generate improved estimations. In addition, recent research has also led to the creation of datasets that were not available when most of these models were first published, and incorporation of these datasets could potentially lead to improvements, i.e. soil moisture, freeze-thaw, improved meteorology, FAPAR, and others.

It may, however, be beneficial to develop regional PEMs that can be combined under a global framework. As global PEMs were intended for application across different vegetation systems, they address only the most fundamental and universal factors governing plant growth [104]. Unique ecophysiological characteristics are therefore not accounted for, which may introduce errors. Current PEMs typically treat the globe similarly in terms of physiology. In an effort to produce global (and in the case of C-Fix and MOD17) operational PEMs with reasonable spatial/temporal resolution, certain assumptions have been made. It is well documented, however, that regional phenomena e.g. permafrost, disturbances, etc have a large influence on NPP. Incorporating these features in regional PEMs under a global framework may lead to improved results.

To date there are few examples of direct empirical validation of PEMs for large territories, due to the evident difficulties of implementing such a procedure. Those that do exist, e.g. [18], rely on eddy covariance measurements and thus the quality of the validation is dependent upon the number and distribution of towers. An alternative approach to validation might include the use of inventory-based NPP datasets where available, which provide complete spatial coverage [105]. Recent efforts to establish a global carbon flux database for forest ecosystems will be helpful [106]. In general, more studies on evaluation of global PEMs are required. Choice of input datasets (e.g. PAR, FAPAR, and others) can have a large impact on the results; therefore more effort is needed here.

Finally, new techniques are being developed to measure rates of photosynthesis and GPP directly, although these are not yet operational. With the recent launch of GOSAT and several proposed ESA/NASA missions, new techniques for carbon flux estimation and PEM calibration and validation will be available in the near future. In the interim, PEMs will likely remain a useful tool in the suite of carbon flux modeling techniques.

## Competing interests

The authors declare that they have no competing interests.

## Authors' contributions

IM conceived the review and drafted the majority of the manuscript. Remaining authors read, edited, and approved the final manuscript.

## References

[B1] Chapin FS, Matson PA, Mooney HA (2002). Principles of Terrestrial Ecosystem Ecology.

[B2] Cramer W, Kicklighter DW, Bondeau A, Moore Iii B, Churkina G, Nemry B, Ruimy A, Schloss AL (1999). Comparing global models of terrestrial net primary productivity (NPP): Overview and key results. Global Change Biology.

[B3] Goetz SJ, Prince SD (1999). Modelling Terrestrial Carbon Exchange and Storage: Evidence and Implications of Functional Convergence in Light-use Efficiency. Advances in Ecological Research.

[B4] Friend AD, Arneth A, Kiang NY, Lomas M, Ogee J, Roedenbeck C, Running SW, Santaren JD, Sitch S, Viovy N (2007). FLUXNET and modelling the global carbon cycle. Global Change Biology.

[B5] Shvidenko A, Nilsson S (2003). A synthesis of the impact of Russian forests on the global carbon budget for 1961-1998. Tellus, Series B: Chemical and Physical Meteorology.

[B6] Beer C, Lucht W, Schmullius C, Shvidenko A (2006). Small net carbon dioxide uptake by Russian forests during 1981-1999. Geophysical Research Letters.

[B7] Stephens BB, Gurney KR, Tans PP, Sweeney C, Peters W, Bruhwiler L, Ciais P, Ramonet M, Bousquet P, Nakazawa T (2007). Weak northern and strong tropical land carbon uptake from vertical profiles of atmospheric CO2. Science.

[B8] Running SW, Nemani RR, Heinsch FA, Zhao M, Reeves M, Hashimoto H (2004). A continuous satellite-derived measure of global terrestrial primary production. BioScience.

[B9] Cihlar J, Denning S, Ahern F, Arino O, Belward A, Bretherton F, Cramer W, Dedieu G, Field C, Francey R (2002). Initiative to quantify terrestrial carbon sources and sinks. EOS, Transactions, American Geophysical Union.

[B10] Lindner M, Lucht W, Bouriaud O, Green T, Janssens I (2004). Specific study on forest greenhouse gas budget. Carbo Europe.

[B11] Running SW, Nemani R, Glassy JM, Thornton PE (1999). MODIS daily photosynthesis (PSN) and annual net primary production (NPP) product (MOD17). Algorithm Theoretical Basis Document (ATBD), Version 30.

[B12] Anderson MC, Norman JM, Meyers TP, Diak GR (2000). An analytical model for estimating canopy transpiration and carbon assimilation fluxes based on canopy light-use efficiency. Agricultural and Forest Meteorology.

[B13] Goetz SJ, Prince SD, Goward SN, Thawley MM, Small J (1999). Satellite remote sensing of primary production: An improved production efficiency modeling approach. Ecological Modelling.

[B14] Verstraeten WW, Veroustraete F, Feyen J (2006). On temperature and water limitation of net ecosystem productivity: Implementation in the C-Fix model. Ecological Modelling.

[B15] Still CJ, Randerson JT, Fung IY (2004). Large-scale plant light-use efficiency inferred from the seasonal cycle of atmospheric CO2. Global Change Biology.

[B16] Ahl DE, Gower ST, Mackay DS, Burrows SN, Norman JM, Diak GR (2004). Heterogeneity of light use efficiency in a northern Wisconsin forest: implications for modeling net primary production with remote sensing. Remote Sensing of Environment.

[B17] Turner DP, Ritts WD, Cohen WB, Maeirsperger TK, Gower ST, Kirschbaum AA, Running SW, Zhao M, Wofsy SC, Dunn AL (2005). Site-level evaluation of satellite-based global terrestrial gross primary production and net primary production monitoring. Global Change Biology.

[B18] Heinsch FA, Zhao M, Running SW, Kimball JS, Nemani RR, Davis KJ, Bolstad PV, Cook BD, Desai AR, Ricciuto DM (2006). Evaluation of remote sensing based terrestrial productivity from MODIS using regional tower eddy flux network observations. IEEE Transactions on Geoscience and Remote Sensing.

[B19] Seixas J, Carvalhais N, Nunes C, Benali A (2007). Ecosystem production modelling in the Iberian Peninsula comparative analysis between MODIS and MERIS datasets. European Space Agency, (Special Publication) ESA SP.

[B20] Jung M, Verstraete M, Gobron N, Reichstein M, Papale D, Bondeau A, Robustelli M, Pinty B (2008). Diagnostic assessment of European gross primary production. Global Change Biology.

[B21] Ruimy A, Jarvis PG, Baldocchi DD, Saugier B (1996). CO2 fluxes over plant canopies and solar radiation: A review. Advances in Ecological Research.

[B22] Gower ST, Kucharik CJ, Norman JM (1999). Direct and indirect estimation of leaf area index, f(APAR), and net primary production of terrestrial ecosystems. Remote Sensing of Environment.

[B23] Glenn EP, Huete AR, Nagler PL, Nelson SG (2008). Relationship between remotely-sensed vegetation indices, canopy attributes and plant physiological processes: What vegetation indices can and cannot tell us about the landscape. Sensors.

[B24] Hilker T, Coops NC, Wulder MA, Black TA, Guy RD (2008). The use of remote sensing in light use efficiency based models of gross primary production: A review of current status and future requirements. Science of the Total Environment.

[B25] Sellers PJ (1987). Canopy reflectance, photosynthesis, and transpiration, II. The role of biophysics in the linearity of their interdependence. Remote Sensing of Environment.

[B26] Monsi M, Saeki T, Schortemeyer M (2005). On the factor light in plant communities and its importance for matter production. Annals of Botany.

[B27] Monteith JL (1972). Solar radiation and productivity in tropical ecosystems. J Appl Ecol.

[B28] Monteith JL (1977). Climate and the efficiency of crop production in Britain. Philos Trans R Soc London, Ser B.

[B29] Jenkins JP, Richardson AD, Braswell BH, Ollinger SV, Hollinger DY, Smith ML (2007). Refining light-use efficiency calculations for a deciduous forest canopy using simultaneous tower-based carbon flux and radiometric measurements. Agricultural and Forest Meteorology.

[B30] Kumar M, Monteith JL (1981). Remote sensing of crop growth. Plants and the Daylight Spectrum.

[B31] Goetz SJ, Prince SD, Small J, Gleason ACR (2000). Interannual variability of global terrestrial primary production: Results of a model driven with satellite observations. Journal of Geophysical Research D: Atmospheres.

[B32] Alexandrov GA, Matsunaga T (2008). Normative productivity of the global vegetation. Carbon Balance Manag.

[B33] Dye DG, Shibasaki R (1995). Intercomparison of global PAR data sets. Geophysical Research Letters.

[B34] Roderick ML (2006). The ever-flickering light. Trends in Ecology & Evolution.

[B35] Gu L, Baldocchi D, Verma SB, Black TA, Vesala T, Falge EM, Dowty PR (2002). Advantages of diffuse radiation for terrestrial ecosystem productivity. Journal of Geophysical Research D: Atmospheres.

[B36] Turner DP, Ritts WD, Cohen WB, Gower ST, Running SW, Zhao M, Costa MH, Kirschbaum AA, Ham JM, Saleska SR, Ahl DE (2006). Evaluation of MODIS NPP and GPP products across multiple biomes. Remote Sensing of Environment.

[B37] Turner DP, Urbanski S, Bremer D, Wofsy SC, Meyers T, Gower ST, Gregory M (2003). A cross-biome comparison of daily light use efficiency for gross primary production. Global Change Biology.

[B38] Gobron N, Verstraete M (2008). ECV T10: Fraction of Absorbed Photosynthetically Active Radiation (FAPAR). Essential Climate Variables.

[B39] Myneni RB, Ross J (1991). Photon-vegetation interactions. Applications in Optical Remote Sensing and Plant Ecology.

[B40] Prince SD (1991). A model of regional primary production for use with coarse resolution satellite data. International Journal of Remote Sensing.

[B41] Schwalm CR, Black TA, Amiro BD, Arain MA, Barr AG, Bourque CPA, Dunn AL, Flanagan LB, Giasson MA, Lafleur PM (2006). Photosynthetic light use efficiency of three biomes across an east-west continental-scale transect in Canada. Agricultural and Forest Meteorology.

[B42] Ruimy A, Kergoat L, Bondeau A (1999). Comparing global models of terrestrial net primary productivity (NPP): Analysis of differences in light absorption and light-use efficiency. Global Change Biology.

[B43] Heinsch FA, Reeves M, Votava P, Kang S, Milesi C, Zhao M (2003). Users Guide: GPP and NPP (MOD17A2/A3) Products NASA MODIS Land Algorithm.

[B44] Ryan MG (1991). Effects of climate change on plant respiration. Ecological Applications.

[B45] Goetz SJ, Prince SD (1998). Variability in carbon exchange and light utilization among boreal forest stands: Implications for remote sensing of net primary production. Canadian Journal of Forest Research.

[B46] Potter C, Klooster S, Tan P, Steinbach M, Kumar V, Genovese V (2005). Variability in terrestrial carbon sinks over two decades: Part 2 - Eurasia. Global and Planetary Change.

[B47] Potter C, Klooster S, Myneni R, Genovese V, Tan PN, Kumar V (2003). Continental-scale comparisons of terrestrial carbon sinks estimated from satellite data and ecosystem modeling 1982-1998. Global and Planetary Change.

[B48] Cao M, Prince SD, Small J, Goetz SJ (2004). Remotely sensed interannual variations and trends in terrestrial net primary productivity 1981-2000. Ecosystems.

[B49] Lafont S, Kergoat L, Dedieu G, Chevillard A, Karstens U, Kolle O (2002). Spatial and temporal variability of land CO2 fluxes estimated with remote sensing and analysis data over western Eurasia. Tellus, Series B: Chemical and Physical Meteorology.

[B50] Veroustraete F, Sabbe H, Rasse DP, Bertels L (2004). Carbon mass fluxes of forests in Belgium determined with low resolution optical sensors. International Journal of Remote Sensing.

[B51] Zhao M, Heinsch FA, Nemani RR, Running SW (2005). Improvements of the MODIS terrestrial gross and net primary production global data set. Remote Sensing of Environment.

[B52] Sasai T, Okamoto K, Hiyama T, Yamaguchi Y (2007). Comparing terrestrial carbon fluxes from the scale of a flux tower to the global scale. Ecological Modelling.

[B53] Sasai T, Ichii K, Yamaguchi Y, Nemani R (2005). Simulating terrestrial carbon fluxes using the new biosphere model BEAMS: Biosphere model integrating Eco-physiological And Mechanistic approaches using Satellite data. J Geophys Res.

[B54] Schloss AL, Kicklighter DW, Kaduk J, Wittenberg U (1999). Comparing global models of terrestrial net primary productivity (NPP): Comparison of NPP to climate and the Normalized Difference Vegetation Index (NDVI). Global Change Biology.

[B55] Reichstein M, Ciais P, Papale D, Valentini R, Running S, Viovy N, Cramer W, Granier A, Ogee J, Allard V (2007). Reduction of ecosystem productivity and respiration during the European summer 2003 climate anomaly: A joint flux tower, remote sensing and modelling analysis. Global Change Biology.

[B56] Verstraeten WW, Veroustraete F, Heyns W, Roey TV, Feyen J (2008). On uncertainties in carbon flux modelling and remotely sensed data assimilation: The Brasschaat pixel case. Advances in Space Research.

[B57] Leith H (1975). Primary productivity in ecosystems: Comparative analysis of global patterns. Unifying Concepts in Ecology.

[B58] Ito A, Sasai T (2006). A comparison of simulation results from two terrestrial carbon cycle models using three climate data sets. Tellus, Series B: Chemical and Physical Meteorology.

[B59] Pan Y, Birdsey R, Hom J, McCullough K, Clark K (2006). Improved estimates of net primary productivity from modis satellite data at regional and local scales. Ecological Applications.

[B60] Hanan NP, Prince SD, Begue A (1995). Estimation of absorbed photosynthetically active radiation and vegetation net production efficiency using satellite data. Agricultural and Forest Meteorology.

[B61] Gitelson AA, Vina A, Verma SB, Rundquist DC, Arkebauer TJ, Keydan G, Leavitt B, Ciganda V, Burba GG, Suyker AE (2006). Relationship between gross primary production and chlorophyll content in crops: Implications for the synoptic monitoring of vegetation productivity. Journal of Geophysical Research D: Atmospheres.

[B62] Rahman AF, Cordova VD, Gamon JA, Schmid HP, Sims DA (2004). Potential of MODIS ocean bands for estimating CO2 flux from terrestrial vegetation: A novel approach. Geophysical Research Letters.

[B63] Sims DA, Rahman AF, Cordova VD, El-Masri BZ, Baldocchi DD, Flanagan LB, Goldstein AH, Hollinger DY, Misson L, Monson RK (2006). On the use of MODIS EVI to assess gross primary productivity of North American ecosystems. Journal of Geophysical Research G: Biogeosciences.

[B64] Grace J, Nichol C, Disney M, Lewis P, Quaife T, Bowyer P (2007). Can we measure terrestrial photosynthesis from space directly, using spectral reflectance and fluorescence?. Global Change Biology.

[B65] Goerner A, Reichstein M, Rambal S (2009). Tracking seasonal drought effects on ecosystem light use efficiency with satellite-based PRI in a Mediterranean forest. Remote Sensing of Environment.

[B66] Mu Q, Zhao M, Heinsch FA, Liu M, Tian H, Running SW (2007). Evaluating water stress controls on primary production in biogeochemical and remote sensing based models. Journal of Geophysical Research G: Biogeosciences.

[B67] Verstraeten WW, Veroustraete F, Wagner W, Van Roey T, Heyns W, Verbeiren S, Sande CJ van der, Feyen J (2007). Impact assessment of remotely sensed soil moisture on ecosystem carbon fluxes across Europe. 2nd International Workshop on Uncertainty in Greenhouse Gas Inventories.

[B68] Beer C, Lucht W, Gerten D, Thonicke K, Schmullius C (2007). Effects of soil freezing and thawing on vegetation carbon density in Siberia: A modeling analysis with the Lund-Potsdam-Jena Dynamic Global Vegetation Model (LPJ-DGVM). Global Biogeochemical Cycles.

[B69] Gifford RM (1994). The global carbon cycle: A viewpoint on the missing sink. Australian Journal of Plant Physiology.

[B70] Hashimoto H, Dungan JL, White MA, Yang F, Michaelis AR, Running SW, Nemani RR (2008). Satellite-based estimation of surface vapor pressure deficits using MODIS land surface temperature data. Remote Sensing of Environment.

[B71] Wagner W, Blöschl G, Pampaloni P, Calvet JC, Bizzarri B, Wigneron JP, Kerr Y (2007). Operational readiness of microwave remote sensing of soil moisture for hydrologic applications. Nordic Hydrology.

[B72] Roderick ML, Farquhar GD, Berry SL, Noble IR (2001). On the direct effect of clouds and atmospheric particles on the productivity and structure of vegetation. Oecologia.

[B73] Alton PB (2008). Reduced carbon sequestration in terrestrial ecosystems under overcast skies compared to clear skies. Agricultural and Forest Meteorology.

[B74] Ibrom A, Oltchev A, June T, Kreilein H, Rakkibu G, Ross T, Panferov O, Gravenhorst G (2008). Variation in photosynthetic light-use efficiency in a mountainous tropical rain forest in Indonesia. Tree Physiology.

[B75] Cook BD, Bolstad PV, Martin JG, Heinsch FA, Davis KJ, Wang W, Desai AR, Teclaw RM (2008). Using light-use and production efficiency models to predict photosynthesis and net carbon exchange during forest canopy disturbance. Ecosystems.

[B76] Lindroth A, Lagergren F, Aurela M, Bjarnadottir B, Christensen T, Dellwik E, Grelle A, Ibrom A, Johansson T, Lankreijer H (2008). Leaf area index is the principal scaling parameter for both gross photosynthesis and ecosystem respiration of Northern deciduous and coniferous forests. Tellus, Series B: Chemical and Physical Meteorology.

[B77] Kimball JS, McDonald KC, Running SW, Frolking SE (2004). Satellite radar remote sensing of seasonal growing seasons for boreal and subalpine evergreen forests. Remote Sensing of Environment.

[B78] Field CB, Randerson JT, Malmström CM (1995). Global net primary production: Combining ecology and remote sensing. Remote Sensing of Environment.

[B79] Ensminger I, Sveshnikov D, Campbell DA, Funk C, Jansson S, Lloyd J, Shibistova O, Oequist G (2004). Intermittent low temperatures constrain spring recovery of photosynthesis in boreal Scots pine forests. Global Change Biology.

[B80] Makela A, Hari P, Berninger F, Hanninen H, Nikinmaa E (2004). Acclimation of photosynthetic capacity in Scots pine to the annual cycle of temperature. Tree Physiology.

[B81] Bartsch A, Kidd RA, Wagner W, Bartalis Z (2007). Temporal and spatial variability of the beginning and end of daily spring freeze/thaw cycles derived from scatterometer data. Remote Sensing of Environment.

[B82] McCallum I, Obersteiner M, Nilsson S, Shvidenko A (2006). A spatial comparison of four satellite derived 1 km global land cover datasets. International Journal of Applied Earth Observation and Geoinformation.

[B83] Fritz S, McCallum I, Schill C, Perger C, Grillmayer R, Achard Fdr, Kraxner F, Obersteiner M (2009). Geo-Wiki.Org: The Use of Crowdsourcing to Improve Global Land Cover. Remote Sensing.

[B84] Sitch S, Smith B, Prentice IC, Arneth A, Bondeau A, Cramer W, Kaplan JO, Levis S, Lucht W, Sykes MT (2003). Evaluation of ecosystem dynamics, plant geography and terrestrial carbon cycling in the LPJ dynamic global vegetation model. Global Change Biology.

[B85] Tansey K, Gregoire JM, Defourny P, Leigh R, Pekel JF, van Bogaert E, Bartholome E (2008). A new, global, multi-annual (2000-2007) burnt area product at 1 km resolution. Geophysical Research Letters.

[B86] Shvidenko AZ, Schepashchenko DG, Vaganov EA, Nilsson S (2008). Net primary production of forest ecosystems of Russia: A new estimate. Doklady Earth Sciences.

[B87] Garrigues S, Lacaze R, Baret F, Morisette JT, Weiss M, Nickeson JE, Fernandes R, Plummer S, Shabanov NV, Myneni RB (2008). Validation and intercomparison of global Leaf Area Index products derived from remote sensing data. Journal of Geophysical Research G: Biogeosciences.

[B88] Fernandes R, Canisius F, Rochdi N, Khurshid S (2008). Integrating a priori in-situ information within satellite based LAI products. Geophysical Research Abstracts.

[B89] Gifford RM (2003). Plant respiration in productivity models: Conceptualisation, representation and issues for global terrestrial carbon-cycle research. Functional Plant Biology.

[B90] Houghton RA (2005). Aboveground forest biomass and the global carbon balance. Global Change Biology.

[B91] Sims DA, Rahman AF, Cordova VD, El-Masri BZ, Baldocchi DD, Bolstad PV, Flanagan LB, Goldstein AH, Hollinger DY, Misson L (2008). A new model of gross primary productivity for North American ecosystems based solely on the enhanced vegetation index and land surface temperature from MODIS. Remote Sensing of Environment.

[B92] Rahman AF, Sims DA, Cordova VD, El-Masri BZ (2005). Potential of MODIS EVI and surface temperature for directly estimating per-pixel ecosystem C fluxes. Geophysical Research Letters.

[B93] Bézy JL, Bensi P, Berger M, Carnicero B, Davidson M, Drinkwater M, Durand Y, Héliere F, Ingmann P, Langen J (2008). ESA future earth observation explorer missions. Proceedings of SPIE - The International Society for Optical Engineering.

[B94] Goetz SJ, Baccini A, Laporte NT, Johns T, Walker W, Kellndorfer J, Houghton RA, Sun M (2009). Mapping and monitoring carbon stocks with satellite observations: A comparison of methods. Carbon Balance and Management.

[B95] Gamon JA, Penuelas J, Field CB (1992). A narrow-waveband spectral index that tracks diurnal changes in photosynthetic efficiency. Remote Sensing of Environment.

[B96] Papale D, Valentini R (2003). A new assessment of European forests carbon exchanges by eddy fluxes and artificial neural network spatialization. Global Change Biology.

[B97] Baldocchi D, Falge E, Gu L, Olson R, Hollinger D, Running S, Anthoni P, Bernhofer C, Davis K, Evans R (2001). FLUXNET: A New Tool to Study the Temporal and Spatial Variability of Ecosystem-Scale Carbon Dioxide, Water Vapor, and Energy Flux Densities. Bulletin of the American Meteorological Society.

[B98] Chapin FS, Woodwell GM, Randerson JT, Rastetter EB, Lovett GM, Baldocchi DD, Clark DA, Harmon ME, Schimel DS, Valentini R (2006). Reconciling carbon-cycle concepts, terminology, and methods. Ecosystems.

[B99] Parton WJ (1993). Observations and modeling of biomass and soil organic matter dynamics for the grassland biome worldwide. Global Biogeochemical Cycles.

[B100] Veroustraete F, Sabbe H, Eerens H (2002). Estimation of carbon mass fluxes over Europe using the C-Fix model and Euroflux data. Remote Sensing of Environment.

[B101] Lloyd J, Taylor JA (1994). On the temperature dependence of soil respiration. Functional Ecology.

[B102] Reichstein M, Rey A, Freibauer A, Tenhunen J, Valentini R, Banza J, Casals P, Cheng Y, Grunzweig JM, Irvine J (2003). Modeling temporal and large-scale spatial variability of soil respiration from soil water availability, temperature and vegetation productivity indices. Global Biogeochemical Cycles.

[B103] Sampson DA, Janssens IA, Curiel Yuste J, Ceulemans R (2007). Basal rates of soil respiration are correlated with photosynthesis in a mixed temperate forest. Global Change Biology.

[B104] Goward SN, Dye DG (1987). Evaluating North American net primary productivity with satellite observations. Advances in Space Research.

[B105] Nilsson S, Shvidenko A, Jonas M, McCallum I, Thomson A, Balzter H (2007). Uncertainties of a regional terrestrial biota full carbon account: A systems analysis. Water, Air, and Soil Pollution: Focus.

[B106] Luyssaert S, Inglima I, Jung M, Richardson AD, Reichstein M, Papale D, Piao SL, Schulze ED, Wingate L, Matteucci G (2007). CO2 balance of boreal, temperate, and tropical forests derived from a global database. Global Change Biology.

[B107] Zhang K, Kimball JS, Zhao M, Oechel WC, Cassano J, Running SW (2007). Sensitivity of pan-Arctic terrestrial net primary productivity simulations to daily surface meteorology from NCEP-NCAR and ERA-40 reanalyses. J Geophys Res.

[B108] Weiss M, Baret F, Garrigues S, Lacaze R (2007). LAI and fAPAR CYCLOPES global products derived from VEGETATION. Part 2: validation and comparison with MODIS collection 4 products. Remote Sensing of Environment.

[B109] Fensholt R, Sandholt I, Rasmussen MS (2004). Evaluation of MODIS LAI, fAPAR and the relation between fAPAR and NDVI in a semi-arid environment using in situ measurements. Remote Sensing of Environment.

